# What are the Antioxidant Status Predictors’ Factors among Male Chronic Obstructive Pulmonary Disease (COPD) Patients?

**DOI:** 10.5539/gjhs.v5n1p70

**Published:** 2012-11-04

**Authors:** Elham Pirabbasi, Mahin Najafiyan, Maria Cheraghi, Suzana Shahar, Zahara Abdul Manaf, Norfadilah Rajab, Roslina Abdul Manap

**Affiliations:** 1 Dietetic Programme, Department of Nursing and Midwifery, Abadan Faculty of Medical Health Sciences, Ahwaz Jundishapur University of Medical Sciences, Ahwaz, Iran; 2 Department of Obstetrics & Gynecology Medical School, Ahwaz Jundishapur University of Medical Sciences, Ahwaz, Iran; 3 Department of Public Health, Faculty of Health Sciences, Ahwaz Jundishapur University of Medical Sciences, Ahwaz, Iran; 4 Dietetic Programme, Center for Health Care Sciences, Faculty of Health Sciences, Universiti Kebangsaan Malaysia, Kuala Lumpur, Malaysia; 5 Biomedical Programme, Faculty of Health Sciences, Universiti Kebangsaan Malaysia, Kuala Lumpur, Malaysia; 6 Department of Medicine, Faculty of Medicine, Universiti Kebangsaan Malaysia, Cheras, Malaysia

**Keywords:** pulmonary disease, antioxidant status, oxidative stress, forced vital capacity, spirometry, plasma vitamin C, malnutrition

## Abstract

Imbalance between antioxidant and oxidative stress is a major risk factor for pathogenesis of some chronic diseases such as chronic obstructive pulmonary disease (COPD). This study aimed to determine antioxidant and oxidative stress status, and also theirs association with respiratory function of male COPD patients to find the antioxidant predictors’ factors. A total of 149 subjects were involved in a cross-sectional study. The study was conducted at two medical centers in Kuala Lumpur, Malaysia. Results of the study showed that plasma vitamin C was low in most of the subjects (86.6%). Total antioxidant capacity was the lowest in COPD stage IV compare to other stages (p < 0.05). Level of plasma vitamin A (p= 0.012) and vitamin C (p= 0.007) were low in malnourished subjects. The predictors for total antioxidant capacity were forced vital capacity (FVC) % predicted and intake of β-carotene (R^2^= 0.104, p= 0.002). Number of cigarette (pack/year) and smoking index (number/year) were not associated with total antioxidant capacity of this COPD population. Plasma oxidative stress as assessed plasma lipid peroxidation (LPO) was only positively correlated with plasma glutathione (p= 0.002). It might be a need to evaluate antioxidant status especially in older COPD patients to treat antioxidant deficiency which is leading to prevent COPD progression.

## 1. Introduction

The lungs are continuously exposed to oxidants generated either endogenously (e.g., released from phagocytes or intracellular oxidants, e.g., from mitochondrial electron transport) or exogenously (e.g., air pollutants or cigarette smoke). The lungs are protected against this oxidative challenge by well-developed enzymatic and non-enzymatic antioxidant systems. Oxidative stress is known to occur when there is an imbalance between oxidants and antioxidants, resulting from either an excess of oxidants and/or depletion of antioxidants (Halliwell, 1996). Smoking is the main etiological factor in COPD. Cigarette smoke contains 10^17^ oxidant molecules per puff, and this, together with a large body of evidence demonstrating increased oxidative stress in smokers and in patients with COPD, has led to the proposal that an oxidant/antioxidant imbalance is important in the pathogenesis of this condition (MacNee, 2000).

Oxidative stress not only produces direct injurious effects in the lungs, but also activates the molecular mechanisms that initiate lung inflammation (Rahman & MacNee, 1998) and may also have a role in many of the processes involved in the complex pathological events that result in COPD. The lung is exposed to oxidants from the environment; however, a large proportion of oxidants produced endogenously in the lungs are by-products of normal cellular metabolism. Mitochondria are the largest source of free radicals as a result of the leaking of an electron from the electron transport chain onto oxygen to form superoxide (Halliwell & Gutteridge, 1999).

Several indicators of oxidative stress, such as hydrogen peroxide exhalation, lipid peroxidation products and degraded proteins, are indeed elevated in COPD patients. As a result, the antioxidant capacity decreases in COPD patients (Boost et al., 2003).

The antioxidants are usually classified as either enzymatic or non-enzymatic and are the primary defenses against reactive oxygen/reactive nitrogen species. The antioxidant enzymes include the superoxide dismutase (SOD) family, catalase, glutathione (GSH) peroxidase, GSH S-transferase and thioredoxin (Halliwell & Gutteridge, 1990). The non-enzymatic antioxidants include low molecular weight compounds, such as GSH, ascorbate, urate, alpha-tocopherol, bilirubin and lipoic acid. Concentrations of these non-enzymatic antioxidants vary in the lungs. Glutathione is more concentrated in epithelial lining fluid compared to plasma (Van der Vliet et al., 1999) and others, such as albumin, are found in high concentrations in serum, but at much lower concentrations in the epithelial lining fluid (Reynolds & Newball, 1974).

The fall in antioxidant capacity of blood from COPD patients should not only be regarded as a reflection of the occurrence of oxidative stress but also as evidence that oxidative stress spreads out to the circulation and can therefore generate a systemic effect such as weight loss and muscle wasting (Boost et al., 2003). Decreased in antioxidant capacity in smokers and patients with COPD, indicate the presence of systemic oxidative stress (Rahman et al., 2000). This systemic effect can be mediated by both oxidative stress and oxidative stress-mediated processes like apoptosis and inflammation (Boost et al., 2003). A diet designed to reduce chronic metabolic stress might form an effective therapeutic strategy in COPD (Boost et al., 2003).

Analysis of data from a representative sample of adults in USA, examined as part of the Second National Health and Nutrition Examination Survey (NHNES II), showed an inverse relationship between both dietary and serum vitamin C and chronic respiratory symptoms. It was suggested that higher dietary intake and serum concentrations of vitamin C had a protective effect against respiratory symptoms. Independent of cigarette smoking, there was an inverse relationship between bronchitis and dietary vitamin C intake (Schwartz & Weiss, 1990). In another study conducted by Schwartz & Weiss in 1994 by using data from NHANES I, found that lower dietary intake of vitamin C was associated with lower FEV_1_ levels in a population survey and lent more support to this view (Schwartz & Weiss, 1994). In NHANES III, the levels of dietary vitamin C, vitamin E, selenium and β-carotene were positively associated with lung function (Hu & Cassano, 2000).

Older studies have been conducted to observe the effect of antioxidants on the respiratory functions of smoking and nonsmoking adults. In 1991, a study of 1502 non-smokers and 1357 smokers with no history of respiratory disease found that fresh fruit consumption in winter, and by implication habitual fruit consumption, was significantly associated with ventilatory functions not only in current smokers but also lifelong nonsmokers. After adjustment for differences in anthropometric measures, socioeconomic status and smoking habits, the forced expiratory volume in one second (FEV_1_) in the group with a low intake of fruit was less (about 80 ml) than when compared with the high intake group (Starchan et al., 1991).

Recent study conducted by Watson and colleagues among smokers and exsmokers with and without COPD after controlling for independent predictors of COPD found that, those with vegetable intake of more or equal 1 portion per day (-1) (93 g) were less likely to have COPD, as were those consuming more or equal 1.5 portions per day (-1) of fruit which was indicated fruit and vegetable consumption is inversely associated with chronic obstructive pulmonary disease and may explain why some smokers do not develop chronic obstructive pulmonary disease (Watson et al., 2002).

A cross sectional study among 2500 adults in Nottinghamshire revealed that not only FEV_1_ was directly associated with habitual vitamin C intake (after adjustment for smoking habits), but also that the effect of vitamin C on FEV_1_ was greater among the older age group. It was suggested that vitamin C had a protective effect on lung function (Britton et al., 1995). In addition to these cross-sectional studies, in a longitudinal study, another group of Dutch investigators examined the relationship between diet and the incidence of chronic non-specific lung disease over a 25 year period, and found that after adjustment for confounding factors, fruit intake was inversely associated with the incidence of lung disease (Miedema et al., 1993). Therefore, the study aimed to evaluate antioxidant and oxidative stress status of male COPD patients regardless to smoking status.

## 2. Methodology

### 2.1 Study Design and Subjects

A cross-sectional study was conducted to determine the antioxidant and oxidative stress status of male COPD patients. Factors associated with antioxidant, oxidative stress status and respiratory functions were also determined. The study was carried out at outpatients’ department (OPD) of the Medical Center of National University of Malaysia and Institute of Respiratory Medicine. The study started from July 2009 until January 2010. Inclusion criteria were male diagnosed with COPD regardless to age, smoking status and COPD stages. The exclusion criteria were inflammatory diseases such as rheumatoid arthritis, tuberculosis and bronchitis. The study was approved by the National University of Malaysia Research Ethics Committee (UKM 1.5.3.5/244/SPP/NN- 056-2009) and also was registered in an open access database trial on 20 January 2012 (ISRCTN18062761). Written consent has been obtained from each subject. A total of 149 subjects who were referred to Medical Center of National University of Malaysia and Institute of Respiratory Medicine were participated in this study. All patients diagnosed with COPD by the physician and who met the inclusion criteria were interviewed to participate. Out of 149 subjects, 52 subjects refused for blood drawing and 44 subjects refused to measure body composition which required fasting at least 8 hours prior to the procedure.

### 2.2 Sample size Calculation and Sampling Method

The formula for sample size calculation was used from below formula (Kish, 1965).

N (subject) = (z ^2^) pq/d^2^

In this formula: the power of study was estimated by 90% and significant level of 0.05 (= d)

z is constant = 1.645 and q = 1-p

P = was the prevalence of malnutrition among COPD patients in Malaysia, which is 12.7% (= 0.127) (Hazlin, 2007).

N= (1.645)^2^ (0.127) (1- 0.127)/(0.05)^2^= 120

N= 120 × 25% (account for attrition) = 150

The convenience (non- probability) sampling method was used. The subjects were selected because they were easiest to recruit for the study and research did not consider selecting subjects that are represented of the entire population.

### 2.3 Measurements

Nutritional status was determined according to anthropometry indicators including, weight, height, mid upper arm circumference (MUAC), triceps skin fold thickness (TSFT) waist circumference (WC) (ISAK, 2001, Lee & Nieman, 2007) calf circumference (CC) (Lohman, et al, 1988) hip circumference (HC) (NHANES III, 1988) and body composition including, fat free mass (FFM) and fat mass (FM). Dietary intake (one day recall and two days record) were also obtained. Subjects were asked to record all foods and drinks consumed for one weekday and one weekend using household measures. The food record was collected during next follow up. Food intake was analysed by Nutritionist- Pro software. Body composition was measured using Maltron body fat analyzer (Maltron, B-F 900, England) for which the subject should fast at least 2-3 hours and urinate 30 minutes prior to the procedure. Lung function was assessed using Spirometry equipment i.e., Spirobank G (AAA MIR, A23-04104545, Italy).

### 2.4 Biochemical Analysis

A total of 10 ml fasting venous blood drawn for measurement of antioxidant (i.e., vitamin A, vitamin E, vitamin C, glutathione and total antioxidant capacity) and oxidative stress status [i.e., lipid peroxidation (LPO)]. In order to measure blood plasma vitamin A and E, Heparinized tube (BD Vacutainer lithium heparin 87 USP units, Ref 367886, USA) was used. Vitamin C (L-ascorbic acid, purity 99%), Vitamin E (± α-tocopherol, purity 95%) and vitamin A (β-carotene, purity 97%) were obtained from Sigma Chemical Company (St. Louis, Mo, USA). Methanol, acetonitrile and tetrahydrofuran were obtained from Merck KGaA (Darmstadt, Germany). All solvents were of HPLC grade. Vitamin A (retinol) and E (α-tocopherol) in serum/plasma were measured by isocratic high performance liquid chromatography (HPLC) with detection at three different wavelengths. The Bio-Rad vitamin A/E serum was used as a calibrator for the Bio-Rad vitamin A/E by HPLC test (Ref 195-5869, GmbH, Germany), for calculating the concentrations of vitamins A or E in patient specimen. The reference range was revised values for the interpretation of serum or plasma retinol and α-tocopherol based on the trials done on 65 apparently healthy Malaysians with a mean age of 52.8 years (range 24 to 76 years) (Tee & Khor, 1995).

In order to measure blood plasma vitamin C, Heparinized tube (BD Vacutainer lithium heparin 87 USP units, Ref 367886, USA) was used. About 1.5 ml venous blood was required to obtain 0.5 ml plasma to measure vitamin C. The plasma was obtained after centrifuging to remove the clot and blood cells. The plasma was mixed with the oxalic acid preservative and wrapped with the aluminum foil to keep at -70°C until run. Vitamin C (L-ascorbic acid, purity: 99%) was obtained from Sigma Chemical Co. (St. Louis, Mo, USA). Methanol, acetonitrile and tetrahydrofuran were obtained from Merck KGaA (Darmstadt, Germany). All solvents were HPLC grade. All the reagents were used without further purification. Deionized water, purified by Milli Q system (Millipore, Milford, MA, USA), was used throughout the procedure (Bin et al., 2004).

Total antioxidant capacity was measured by the ferric reducing antioxidant power (FRAP) assay (Benzie & Strain, 1999). In order to measure plasma glutathione, EDTA tube (BD Vacutainer K2 EDTA10.8 mg, Ref 367863, USA) was used. About 1 ml fasting venous blood was extracted to 0.5 ml plasma to measure glutathione. The sample was stored at -20°C up to -70°C until analyzed. The Calbiochem® GSH Assay Kit II (Cat. No. 354103) was used to determine the concentration of GSH in human plasma. The Calbiochem® Lipid Hydroperoxide (LPO) Assay Kit measured lipid hydroperoxide directly utilizing redox reactions with ferrous ions. The EDTA plasma was used in this assay.

### 2.5 Statistical Analysis

Data were analyzed using Statistical Package for Social Sciences (SPSS) version 16. A Kolmogorov-Smirnov test was performed prior to statistical analysis in order to examine the normality of the variables. In order to find the relationship between parametric variable, Pearson Correlation test and for non parametric variable Spearman Coefficient Correlation were applied. In addition, to compare means of more than two groups with one normally continuous variable, One-Way ANOVA test and for non parametric variable Kruskal-Wallis test were performed. In order to find out the association between 2 grouping variable (2_2 table) Chi-squared test was performed by considering odds ratio and 95% confidence interval. The predictors for antioxidant and oxidative stress status were determined using Multiple Regression to find the best model. The step- wise method was used to assess the accuracy and stability of the model. The entire statistic test p value was 2-sided significant at the level of 0.05.

## 3. Results

In order to determine antioxidant and oxidative stress of the subjects, blood plasma was analysed to measure the level of common and important antioxidant in body such as vitamin A, E, C, glutathione (GSH) and total antioxidant capacity (TAC). Oxidative stress status was assessed by measuring plasma lipid peroxidation (LPO). Results revealed that plasma vitamin C was low in most of the subjects (86.6 %). The level of plasma vitamin A and E was normal in 71.1% and 61.9% of the subjects respectively (Table 1). A higher percentage of the subjects in younger age group had low plasma vitamin A and E. However, most of the subjects in older age group (88.1%) had low plasma vitamin C as compared to the younger age group (83.3%) (Table 1). There was not found any statistical significant difference between antioxidant status and both age groups of the subjects. The level of total antioxidant capacity was the lowest in COPD stage IV and the highest in stage I. The mean and standard deviation of total antioxidant capacity reduced with the advanced stage of COPD (p < 0.05) (Table 2). A higher percentage of the subjects with mild to moderate COPD (stage I and II) had low plasma vitamin C status (94.9%) as compared to severe COPD (stage III and IV) (81.0%) (p < 0.05) (Table 3). A higher percentage of the subjects with malnutrition (FFMI < 16 kg/m^2^) had low plasma vitamin A (43.6%) as compared to the non malnourished (19.0%) (p < 0.05). However, inverse observation was noted for vitamin C, as shown in Table 4.

**Table 1:** Plasma antioxidant status of subjects according to age group (Expressed as number and %) (n=97)]

Variable (Unit)	35-50 yrs (n= 29)	≥ 60 yrs (n= 68)	Total (n=97)	p
	n (%)	n (%)	n (%)	
**Vitamin E (μmol/L)**				
Normal (18.58- 67.34)	14 (48.3)	46 (67.6)	60 (61.9)	0.072
Low (< 18.58)	15 (51.7)	22 (32.4)	37 (38.1)	
**Vitamin A (μmol/L)**				
Normal (1.05- 3.84)	20 (69.0)	49 (72.1)	70 (71.1)	0.75
Low (< 1.05)	9 (31.0)	19 (27.9)	28 (28.9)	
**Vitamin C (mg/dl)**				
Normal (0.4- 1.5)	5 (16.7)	8 (11.9)	13 (13.4)	0.52
Low (< 0.4)	25 (83.3)	59 (88.1)	84 (86.6)	

Not significant using Pearson Chi- Square test

**Table 2:** Concentration of Plasma Antioxidant and Oxidative Stress of Subjects According to COPD Staging (n= 97)

Variable (Unit)	Stage I (n= 10)	Stage II (n= 29)	Stage III (n=40)	Stage IV (n=18)	p
	Mean rank	Mean rank	Mean rank	Mean rank	
					
Vitamin E (μmol/L)	53.25	45.36	51.09	47.86	0.810^a^
Vitamin A (μmol/L)	49.25	46.21	54.48	41.19	0.364^a^
Vitamin C (mg/dl)	43.80	44.07	49.69	58.31	0.358^a^
Glutathione (μM)	43.45	56.14	46.96	54.11	0.422^a^
Total Antioxidant Capacity (μmol/L)	504.79 ± 331.50	408.67± 189.76	314.69± 129.22	310.96 ± 149.78	0.040^b^
Lipid Peroxidation (μM)	36.65	47.57	52.91	44.33	0.354^a^

Not significant using

a Kruskal- Wallis test, p < 0.05 significant using

b One –Way ANOVA (data presented as Mean± SD)

**Table 3:** Plasma Antioxidant Status of Subjects According to the Severity of COPD (Presented as Number and %) (n=97)

Variable (Unit)	Stage I & II (n= 39)	Stage III&IV (n= 58)	P
	n (%)	n (%)	
**Vitamin E (μmol/L)**			
Normal (18.58- 67.34)	24 (61.5)	36 (62.1)	0.96
Low (< 18.58)	15 (38.5)	22 (37.9)	
**Vitamin A (μmol/L)**			
Normal (1.05- 3.84)	27 (69.2)	42 (72.4)	0.73
Low (< 1.05)	12 (30.8)	16 (27.6)	
**Vitamin C (mg/dl)**			
Normal (0.4-1.5)	2 (5.1)	11 (19.0)	0.049*
Low (< 0.4)	37 (94.9)	47 (81.0)	

* p <0.05 is significant using Pearson Chi- Square test

**Table 4:** Plasma Antioxidant Status of Subjects According to FFMI [Expressed as Number and % (n=97)]

Variable (Unit)	FFMI < 16 kg/m^2^ (n= 39)	FFMI ≥ 16 kg/m^2^ (n=58)	P
	n(%)	n(%)	
**Vitamin E (μmol/L)**			
Normal (18.58- 67.34)	22 (56.4)	38 (65.6)	0.400
Low (< 18.58)	17 (43.6)	20 (34.4)	
**Vitamin A (μmol/L)**			
Normal (1.05- 3.84)	22 (56.4)	47 (81.0)	0.012*
Low (< 1.05)	17 (43.6)	11 (19.0)	
**Vitamin C (mg/dl)**			
Normal (0.4-1.5)	10 (25.0)	3 (5.3)	0.007*
Low (< 0.4)	30 (75.0)	54 (94.7)	

* p < 0.05 significant using Fisher Exact test

Assessment of food analysis showed that intake of vitamin A was 30.2% (n= 45) was lower than Malaysian RNI whereas intake of vitamin E was 100% (n=149) lower than Malaysian RNI among the subjects. Intake of vitamin C was 94% (n=140) lower than Malaysian RNI (Table was not shown).

Nutritional status i.e., waist circumference and waist/hip ratio was positively correlated with plasma vitamin E of the subjects. Plasma vitamin A was also significantly correlated with anthropometry parameters including BMI, MUAC, FFMI, and calf circumference, waist circumference and waist/hip ratio. Plasma vitamin C was negatively correlated with hip circumference (r = -0.206, p < 0.05). Plasma glutathione and total antioxidant capacity were not correlated with anthropometry or body composition of COPD subjects (Table 5). It seems that plasma vitamin E and A status increased with higher values of anthropometric indicators or vice versa.

**Table 5:** Correlation between Plasma Antioxidant and Nutritional Status of Subjects (n= 97)

Variable	Plasma vitamin A (μmol/L)		Plasma vitamin E (μmol/L)		Plasma vitamin C (mg/dl)		Plasma Glutathione (μM)		Plasma Total Antioxidant Capacity (μmol/L)	

	R	p	r	p	r	p	R	p	r	p
Body Mass Index (kg/m^2^)	0.28	0.006^*a^	0.194	0.057^a^	-0.155	0.130^a^	-0.002	0.988^a^	0.161	0.126^b^
Triceps Skin Fold Thickness (mm)	-0.014	0.892^a^	0.143	0.163^a^	0.065	0.524	-0.068	0.506^a^	0.070	0.507^b^
Mid Upper Arm Circumference (cm)	0.22	0.028^*a^	0.195	0.055^a^	-0.185	0.069^a^	0.010	0.920^a^	0.161	0.126^b^
Calf Circumference (cm)	0.21	0.033^*a^	0.161	0.115^a^	-0.149	0.146^a^	0.014	0.894^a^	0.184	0.081^b^
Waist Circumference (cm)	0.371	0.001^*a^	0.274	0.007^*a^	-0.177	0.082^a^	-0.069	0.504^a^	0.075	0.479^b^
Hip Circumference (cm)	0.17	0.089^a^	0.158	0.122^a^	-0.206	0.043^*a^	-0.024	0.814^a^	0.127	0.229^b^
Waist/hip ratio	0.39	0.001^*a^	0.258	0.011^*a^	-0.088	0.394^a^	-0.097	0.343^a^	-0.013	0.903^b^
Handgrip (kg)	0.049	0.631^a^	0.032	0.753^a^	-0.091	0.373^a^	-0.046	0.655^a^	0.088	0.408^b^
Lean Mass Weight (kg)	0.19	0.053^a^	0.126	0.218^a^	0.069	0.465^a^	0.007	0.946^a^	0.082	0.441^b^
Lean Mass %	-0.149	0.147^a^	-0.148	0.149^a^	0.069	0.503^a^	-0.025	0.806^a^	-0.087	0.412^b^
Body Fat Mass (kg)	0.179	0.080^a^	0.134	0.189^a^	-0.104	0.310^a^	-0.019	0.856^a^	0.098	0.354^b^
Body Fat %	0.128	0.214^a^	0.134	0.193^a^	-0.046	0.653^a^	0.069	0.506^a^	0.053	0.617^b^
Fat Free Mass Index (kg/m^2)^	0.201	0.049^*a^	0.101	0.323^a^	-0.087	0.395^a^	-0.014	0.894^a^	0.088	0.405^b^

*a p < 0.05 significant using Spearman Correlation Coefficient test

^*b^ p < 0.05 significant using Pearson Correlation test

In order to find the predictors for antioxidant status i.e., total antioxidant capacity (TAC), a Univariate analysis was performed to find out the significant association. Variables found to be significant in the Univariate analysis were entered into Multiple Linear Regression analysis to determine the best predictors. The highest adjusted R square was the best model predictors. Using Univariate analysis, it was found that β-carotene intake, FVC% predicted and plasma vitamin E were associated with plasma total antioxidant capacity (Table was not shown). Multiple Linear Regression analysis indicated that FVC% predicted and intake of β-carotene were the predictors of plasma total antioxidant capacity (R^2^ = 0.104, p < 0.05) (Table 6). Univariate analysis indicated that fat% and plasma glutathione were associated with oxidative stress status or lipid peroxidation (LPO) (Table was not shown). Multiple Linear Regression analysis indicated that only plasma glutathione was the predictor of LPO (R^2^ = 0.045, p < 0.05) (Table 7). High concentration of plasma glutathione was associated with high level of LPO.

**Table 6:** Multiple regression model predicting plasma antioxidant status of COPD subjects

Independent Variable	Plasma total antioxidant capacity (R^2^=0.104, F=6.530, p= 0.002)		

	t	B	P
FVC% predicted	3.134	2.736	0.002
Intake of β-carotene (μg/day)	-2.126	-0.010	0.036

p < 0.05 significant using Multiple Linear Regression Analysis

Adjusted for Plasma total antioxidant capacity: Intake of β-carotene, FVC% predicted and plasma vitamin E

**Table 7:** Multiple regression model predicting plasma oxidative stress (LPO) of COPD subjects

Independent Variable	Plasma LPO (R^2^= 0.045, F= 5.471, p= 0.021)		

	t	B	p
Plasma glutathione (μM)	2.339	0.159	0.021

p < 0.05 significant using Multiple Linear Regression Analysis Adjusted for Plasma LPO: Fat % and plasma glutathione

## 4. Discussion

With respect to antioxidant intake, 30.2 % of the subjects had inadequate intake of vitamin A, vitamin E (100%) and vitamin C (94%) respectively, which the deficiency was more seen in older subjects aged 60 years and above. The subjects consumed less fresh vegetable and fruits through their diet. Similar finding was reported by Lin et al. (2010) among COPD patients in Taiwan. They reported that intakes of vitamin C and several carotenoids were lower in the COPD group as compared to healthy group.

The plasma antioxidant status such as plasma vitamin C was low in most of the study subjects (86.6%). The subjects with mild to moderate COPD (stage I and II) had low level of plasma vitamin E (38.5%), vitamin A (30.8%) and vitamin C (94.9%) as compared to severe COPD group (stage III and IV). The plasma concentration of vitamin A (43.6%) and vitamin E (43.6%) was low in malnourished subjects perhaps due to low consumption of fruits and fresh vegetables through their diet. Lin et al, (Lin et al, 2010) also noted that COPD patients had significantly lower plasma concentrations of vitamins A, C, and E; α- and β-carotene; and total carotenoids, they concluded that COPD patients in Taiwan have lower levels of antioxidant in their plasma and diet than do healthy people. Intakes of vitamin C and carotenoids were correlated with dietary habits. The results of the present study also was supported by the study conducted by Khan that showed a diet poor in antioxidants among COPD patients was common, with 25% (selenium), 45% (vitamin C), 90% (vitamin E), 55% (vitamin A), and 70% (vitamin D) deficiencies (Khan, 2010).

Furthermore, a higher plasma total antioxidant capacity was associated with a better respiratory function i.e., FVC % predicted. Similar finding was found in a study among COPD patients by Ness et al, that plasma vitamin C was positively correlated, after adjustment for age and height, with lung function in men (Ness et al, 1996). Vitamin C is water soluble antioxidant but vitamin C can be a pro-oxidant and oxidative stress biomarker when concentration in body is high. Higher oxidative stress and free radicals lead to hypercapnia and decreased lung function and severity of disease. The results of the current study also were supported by another study in USA. They reported that antioxidants such as serum β-cryptoxanthin, lutein/zeaxanthin, and retinol, and dietary β-carotene, β-cryptoxanthin, lutein/zeaxanthin, vitamin C, and lycopene were positively associated with FEV_1_% (p < 0.05, for all associations). Serum vitamins β-cryptoxanthin, lutein/zeaxanthin, and lycopene, and dietary β-cryptoxanthin, β-carotene, vitamin C, and lutein/zeaxanthin were positively associated with FVC% (p < 0.05, for all associations). Erythrocytic glutathione was negatively associated with FEV_1_%, while plasma thiobarbituric acid reactive substances (TBARS) were negatively associated with FVC% (p< 0.05) (Schünemann & Ochs-Balcom, 2006).

Fat percentage and plasma glutathione were also associated with lipid peroxidation (LPO). Lipid peroxidation results in the formation of highly reactive and unstable hydroperoxide of both saturated and unsaturated fat. Antioxidant glutathione also is able to be pro-oxidant like vitamin C when concentration in body is high. Therefore, high intake of fat and high concentration of glutathione leads to increased LPO.

In year 2000, Gosker et al. (2000) in Netherlands found that antioxidant system among COPD patients may be exposed by elevated levels of oxidative stress. Biopsies from the vastus lateralis of 21 patients with COPD and 12 healthy age-matched controls were analyzed. Total antioxidant capacity, vitamin E, glutathione, and uric acid levels were determined and the enzyme activities of superoxide dismutase, glutathione reductase, glutathione peroxidase, and glutathione-S-transferase were measured. Malondialdehyde was measured as an index of lipid peroxidation (LPO). The total antioxidant capacity and the uric acid levels were markedly higher in COPD patients as compared to healthy controls (25%, p = 0.006 and 24%, p = 0.029, respectively). Glutathione-S- transferase activity was also increased (35%, p = 0.044) in patients as compared to healthy subjects. Vitamin E level was lower in COPD patients as compared to controls (p < 0.05). The malondialdehyde level was not different between two groups. The muscle total antioxidant capacity was increased in patients with COPD. Also level of vitamin E reduced and glutathione-S-transferase activity increased but level of lipid peroxidation products was normal (Gosker et al., 2000).

Lipid peroxidation (LPO) can interact with enzymatic or nonenzymatic antioxidants such as glutathione or decompose after reacting with metal ions or iron-containing proteins, forming hydrocarbon gases and unsaturated aldehydes as by-products (Repine et al., 1997). This finding supports result of this study that plasma antioxidant glutathione is a predictor for lipid peroxidation.

## 5. Conclusion

In summary, all these studies appear to show a consistent pattern, associated with better lung function and relative freedom from chronic lung disease with intake of nutrients high in antioxidants (fresh fruits). Therefore, antioxidant assessment among COPD patients might be beneficial effect to prevent progression of air flow limitation in COPD patients. It seems that antioxidant supplementation may be a need in the subjects with antioxidant deficiency.
